# Deep Learning-Based Detection of Depression and Suicidal Tendencies in Social Media Data with Feature Selection

**DOI:** 10.3390/bs15030352

**Published:** 2025-03-12

**Authors:** İsmail Baydili, Burak Tasci, Gülay Tasci

**Affiliations:** 1 Department of Audiovisual Techniques and Media Production, Vocational School of Technical Sciences, Fırat University, Elazig 23119, Turkey; ibaydili@firat.edu.tr; 2 Vocational School of Technical Sciences, Firat University, Elazig 23119, Turkey; 3 Department of Psychiatry, Elazig Fethi Sekin City Hospital, Elazig 23280, Turkey

**Keywords:** depression and suicidal tendencies, depression detection, suicidal ideation, social media analysis, pre-trained language models, feature selection, Support Vector Machines, deep learning, mental health

## Abstract

Social media has become an essential platform for understanding human behavior, particularly in relation to mental health conditions such as depression and suicidal tendencies. Given the increasing reliance on digital communication, the ability to automatically detect individuals at risk through their social media activity holds significant potential for early intervention and mental health support. This study proposes a machine learning-based framework that integrates pre-trained language models and advanced feature selection techniques to improve the detection of depression and suicidal tendencies from social media data. We utilize six diverse datasets, collected from platforms such as Twitter and Reddit, ensuring a broad evaluation of model robustness. The proposed methodology incorporates Cumulative Weight-based Iterative Neighborhood Component Analysis (CWINCA) for feature selection and Support Vector Machines (SVMs) for classification. The results indicate that the model achieves high accuracy across multiple datasets, ranging from 80.74% to 99.96%, demonstrating its effectiveness in identifying risk factors associated with mental health issues. These findings highlight the potential of social media-based automated detection methods as complementary tools for mental health professionals. Future work will focus on real-time detection capabilities and multilingual adaptation to enhance the practical applicability of the proposed approach.

## 1. Introduction

The rapid expansion of digital communication technologies has fundamentally transformed the way individuals interact, share information, and express emotions. Social media platforms have become integral to modern life, allowing users to communicate instantly, access diverse content, and participate in large-scale discussions (Yujie et al., 2022). Unlike traditional media, which primarily facilitate one-way information dissemination, social media enables real-time, bidirectional engagement, making individuals active contributors rather than passive consumers (Bingham & Conner, 2010). According to recent global statistics, approximately 5.22 billion people actively use social media, reflecting an annual increase of 250 million users (We Are Social, 2024). The rise of these platforms has created both opportunities and challenges, particularly concerning mental health, as individuals increasingly use social media to disclose personal struggles, emotions, and distress (Bin Abdullah, 2023; Van Dijck & Poell, 2013).

Depression and suicidal tendencies have emerged as significant public health concerns, with global suicide rates ranking among the leading causes of mortality, particularly among young adults (Kietzmann et al., 2011). The World Health Organization (WHO) identifies suicide as the fourth leading cause of death among individuals aged 15–29, emphasizing the need for effective early detection and intervention strategies (Kaplan & Haenlein, 2010).Traditional psychiatric evaluations rely on self-reported assessments, structured interviews, and clinical observations, which, while valuable, are often time-consuming and may fail to capture real-time psychological fluctuations (Li et al., 2022). In contrast, digital footprints left by individuals on social media provide an opportunity for large-scale, automated mental health monitoring. Studies indicate that individuals experiencing depression or suicidal thoughts exhibit distinct linguistic patterns, behavioral changes, and social withdrawal in their online interactions, which can be analyzed using artificial intelligence and natural language processing techniques (Balkin, 2017).

Despite the growing interest in the AI-assisted detection of depression and suicidal tendencies, existing methodologies face several limitations. Many studies rely on data obtained from a single social media platform such as X or Reddit, which restricts their generalizability across different populations and linguistic contexts (Dahlgren, 2009). High-dimensional textual data present challenges in feature selection, as redundant and irrelevant features often lead to overfitting and reduced interpretability (Baydili et al., 2025). Additionally, class imbalance remains a critical issue, as datasets related to depression and suicidal tendencies frequently contain a disproportionately low number of high-risk cases, making it difficult for models to detect less common but clinically significant instances (Sheldon et al., 2019). Another challenge is the lack of real-time application, as most existing models focus on retrospective analysis rather than proactive intervention, limiting their practical use in mental health monitoring systems (Hammad & Awed, 2023). Recent studies have demonstrated the effectiveness of NLP and deep learning techniques in detecting mental health conditions through social media data. Transformer-based architectures such as BERT, RoBERTa, and DeBERTa have been widely adopted for their ability to capture complex linguistic patterns associated with depression and suicidal ideation, achieving classification accuracies above 98% (Akintoye et al., 2024; Zhang et al., 2024). Additionally, hybrid deep learning models, such as CNN-BiLSTM with attention mechanisms, have further improved classification performance, reaching 94.29% accuracy in similar tasks (Bhuiyan et al., 2025).

Despite these advancements, challenges such as generalizability across platforms, real-time applicability, and ethical considerations remain unaddressed. Most prior studies focused on data from a single platform, limiting models’ robustness when applied to different linguistic and social contexts (Mansoor & Ansari, 2024; Zhang et al., 2024). Moreover, explainability remains a concern, as black-box AI models lack transparency, making it difficult for mental health professionals to trust their outputs (Bhuiyan et al., 2025; Islam et al., 2023). Addressing these gaps requires a more comprehensive approach that integrates multiple datasets, ensures model interpretability, and leverages ensemble learning to enhance detection accuracy and robustness.

To address these challenges, this study proposes a novel AI-driven framework that integrates multiple pre-trained language models, including BERT, RoBERTa, and ClinicalBERT, with Cumulative Weight-based Iterative Neighborhood Component Analysis (CWINCA) for feature selection and Support Vector Machines (SVMs) for classification. The proposed approach enhances the classification performance by optimizing feature selection while maintaining model interpretability. By incorporating an iterative majority voting (IMV) mechanism, the model improves robustness and mitigates class imbalance issues. Unlike previous studies, this research evaluates six diverse datasets, ensuring a broader assessment of model generalizability and real-world applicability.

Beyond its technical contributions, this study also explores the clinical implications of the AI-driven detection of depression and suicidal tendencies. If a user’s online activity indicates suicidal tendencies or severe depressive symptoms, an automated system could facilitate timely intervention by alerting mental health professionals and displaying crisis support options, or via integration with digital mental health services. As social media platforms continue to serve as primary spaces for emotional expression, leveraging AI for early risk detection presents an opportunity to enhance suicide prevention and mental health care (Braghieri et al., 2022). However, ethical considerations related to privacy, consent, and potential misclassification must also be addressed to ensure the responsible deployment of AI in mental health applications (Sun, 2023).

By extracting relevant attributes from social media data, this study aims to identify risk factors associated with these conditions, thereby facilitating more effective intervention strategies.

The remainder of this paper is structured as follows. Section 2 reviews the existing literature on detecting depression and suicidal tendencies using social media data, highlighting key methodologies and challenges. Section 3 describes the datasets used in this study, while Section 4 details the proposed methodology, including feature extraction, feature selection, and classification techniques. Section 5 presents experimental results, followed by a discussion in Section 6. Finally, Section 7 concludes the paper and outlines directions for future research.

## 2. Literature Review

The detection of depression and suicidal tendencies from social media data has gained increasing attention due to the growing digital footprint of individuals experiencing psychological distress.

The unobtrusive detection of mental health disorders through social media analysis has gained increasing attention as an alternative to self-reported psychiatric assessments. Previous studies have explored a variety of NLP and deep learning approaches to identify distress-related patterns in textual data. For example, transformer-based models such as RoBERTa and DeBERTa have been found to outperform traditional classifiers, achieving up to 99.6% accuracy in detecting suicidal ideation (Akintoye et al., 2024; Zhang et al., 2024). Furthermore, hybrid deep learning models, such as CNN-BiLSTM with attention mechanisms, have demonstrated robust performances, reaching 94.29% accuracy in classifying depression symptoms (Bhuiyan et al., 2025).

Numerous studies have explored various machine learning and deep learning techniques to classify mental health-related content, with approaches ranging from traditional feature-based methods to state-of-the-art transformer models (Bokolo & Liu, 2023; Boulouard et al., 2022; J. Liu et al., 2022). However, despite advances in artificial intelligence, major challenges persist, including issues of generalizability, real-time applicability, feature interpretability, and data biases.

### 2.1. Deep Learning-Based Approaches

Deep learning models have demonstrated strong performances in analyzing social media data for depression and suicidal ideation detection. Qorich and El Ouazzani (2024) applied CNN, BiLSTM, and C-BiLSTM models with embedding techniques such as Word2Vec and FastText, achieving 97.69% accuracy with a GPT-based model. However, their study was confined to Reddit data, limiting its applicability to other platforms. Bauer et al. (2024) used BERT-based embedding to examine suicide risk factors in 2.9 million Reddit posts, categorizing user posts by mood, stress severity, and support-seeking behavior. Despite its success, the study lacked a longitudinal analysis and was restricted to Reddit, raising concerns about generalizability across diverse social media platforms.

Bokolo and Liu (2023) compared machine learning and transformer-based techniques for depression detection on a dataset of 632,000 tweets. Their results showed that RoBERTa outperformed other models, with an accuracy of 98.1%. However, the dataset was originally designed for sentiment analysis rather than direct mental health diagnostics, which may have introduced biases in depression labeling. Similarly, J. Liu et al. (2022) proposed an ensemble method combining feature fusion techniques for suicidal ideation detection. While effective, their model required extensive feature engineering, which may not scale efficiently for real-time applications.

### 2.2. Explainable AI and Feature Engineering Approaches

To enhance model interpretability, researchers integrated explainable artificial intelligence (XAI) techniques with deep learning. Malhotra and Jindal (2024) used SHAP and LIME with a BERT-based model to analyze depressive and suicidal behaviors, achieving an F1 score of 88.5%. However, data labeling inconsistencies and the challenge of handling unlabeled data reduced the reliability of their findings. Bao et al. (2024) leveraged BDI-II and PsySym datasets for depression detection, demonstrating that explainable AI models improve classification performance. Yet, their study was constrained to structured datasets, limiting applicability to unstructured, real-world social media data.

### 2.3. Hybrid and Ensemble Learning Approaches

Ahmed et al. (2024) introduced an ensemble model, integrating BERT, BERTweet, and ALBERT, to classify depression severity into four levels. The model outperformed previous approaches to the DEPTWEET dataset (AUC-ROC: 97.59%) but struggled with class imbalance and differentiation between mild and moderate depression. Khowaja et al. (2024) proposed an emotionally aware coder and fuzzy-based adversarial learning network (EAC-Net) for depression detection. The model, incorporating MentalRoBERTa and Emotion2Vec, achieved up to 3.64% improvement in F1 scores over baseline models. However, its reliance solely on textual data was a significant limitation. Bhaumik et al. (2023) developed an AI tool called MindWatch, using LLMs like ALBERT and Bio-Clinical BERT to detect suicidal thoughts. On a dataset of 2.32 million Reddit posts, the ALBERT model achieved an AUC of 0.98 with over 92% accuracy and F1 scores. Limitations included data biases and a lack of generalization, common to LLMs. X. Lan et al. (2024) developed the DORIS system to detect depression symptoms in social media posts. This system used DSM-5 criteria for automatic labeling and analyzed “mood courses” in emotionally intense texts. A GBT classifier, combining traditional methods with large language models, outperformed the best existing methods with an AUPRC score of 0.8134, a 0.036 improvement. The study’s limitations included its focus on a unique dataset restricted to depression detection. Farruque et al. (2024) proposed a semi-supervised learning approach, using BERT-based models to classify depressive symptoms in one of the largest datasets labeled by clinical experts. The model surpassed prior approaches with a macro F1 score of 45% and a weighted F1 score of 56%. Limitations included small sample sizes and low accuracy rates for specific symptom labels.

### 2.4. Real-Time and Interactive Models

The applicability of depression and suicide risk detection models in real-time settings remains a significant challenge. Dai et al. (2024) developed an AI framework for real-time suicide risk prediction using the Sina Microblog platform, achieving a 4.12% improvement in accuracy through a human-in-the-loop feedback mechanism. However, the model’s reliance on annotated data and its computational demands hindered scalability.

Many deep learning-based approaches require extensive preprocessing and feature extraction, leading to latency issues that obstruct real-time deployment. Additionally, models trained on static datasets often struggle to generalize to the rapidly evolving nature of social media content, reducing their effectiveness in early intervention systems. Addressing these limitations necessitates the development of adaptive models that can dynamically update based on new data while balancing accuracy and computational efficiency.

### 2.5. Deficiencies in Prior Research

Despite advancements in the AI-driven detection of depression and suicidal tendencies using social media data, several challenges remain that limit the effectiveness and applicability of existing models.

One key limitation is the reliance on data from a single social media platform, which restricts the adaptability of models across different linguistic and cultural contexts. The lack of cross-platform validation reduces robustness, making it difficult to generalize findings to diverse user populations.

Another challenge is the handling of high-dimensional feature spaces. Without effective feature selection techniques, models often retain redundant or irrelevant information, increasing computational complexity and reducing interpretability (Ince et al., 2025; Tuncer et al., 2024). This makes it harder to derive meaningful insights from the extracted features, limiting the practical use of AI-based detection tools.

Real-time detection and intervention also remain problematic. Many existing approaches focus on retrospective analysis, which delays potential mental health interventions. Additionally, static datasets may not fully capture evolving linguistic patterns in social media discourse, affecting model adaptability and performance over time.

Class imbalance presents another significant issue, particularly in datasets where high-risk cases are underrepresented (AlHamed et al., 2022). This imbalance can lead to biased predictions, where less frequent but clinically significant cases are overlooked. Addressing this issue requires improved data balancing techniques and adaptive learning strategies to enhance model sensitivity and fairness.

To improve the detection of depression and suicidal tendencies through social media analysis, future research should focus on developing more adaptable and computationally efficient frameworks. Enhancing cross-platform validation, refining feature selection, integrating real-time adaptability, and addressing class imbalance will contribute to the development of more reliable and effective AI-driven mental health monitoring systems.

### 2.6. Novelties and Contributions

By analyzing social media data, this study proposes a new AI-based framework that can be used to detect suicidal ideation and depression. The main contributions of this study to related literature are summarized below:

- For the first time, an ensemble of diverse pre-trained language models, including BERT, RoBERTa, ALBERT, ClinicalBERT, BioBERT, DistilBERT, ELECTRA, and XLNet, is integrated to extract contextual features from social media data. This ensemble-based strategy leverages the strengths of individual models to generate robust and comprehensive feature representations.
- While multiple pre-trained language models have been employed in previous studies for psychiatric disorder detection, our approach introduces several key advancements. First, we integrate a diverse set of models, ensuring robust contextual representation across different linguistic domains. Second, we implement Cumulative Weight-based Iterative Neighborhood Component Analysis (CWINCA) to optimize feature selection, eliminating redundant and less relevant features while enhancing interpretability. Third, our model utilizes an iterative majority voting strategy, aggregating predictions from different classifiers to improve overall accuracy and robustness. These innovations allow our framework to outperform existing approaches in terms of generalizability, efficiency, and real-time applicability.
- Cumulative Weight-based Iterative Neighborhood Component Analysis (CWINCA) is employed to identify meaningful and significant features from high-dimensional text data. This technique effectively eliminates redundant features, resulting in substantial improvements in classification performance.
- Predictions from different language models and classifiers are combined using an iterative majority voting method. This approach enhances the overall performance of the framework, significantly improving accuracy rates.
- The model’s generalizability and effectiveness are assessed across six diverse social media datasets: Suicidal Ideation Detection, Depression Detection from Reddit Posts, Mental Health Corpus, Twitter US Airline Sentiment, Suicide and Depression Detection, and Sentiment140. The comprehensive assessment revealed uniform performance across multiple problem areas.

The suggested system attained superior performance, especially when employing a Support Vector Machine (SVM) as the classifier. It achieved an accuracy of 99.96% on the Suicide and Depression Detection dataset, 98.9% on Depression: Reddit Dataset (Cleaned), and 96.5% on Sentiment140 dataset, markedly better than the performance of current methodologies in the literature. These contributions highlight the revolutionary capacity of artificial intelligence and natural language processing methods in identifying and analyzing psychiatric diseases. Moreover, the results underscore the significance of social media data as a resource for public health research. This research establishes a robust basis for subsequent efforts focused on creating early identification and intervention strategies, hence enhancing mental health solutions.

## 3. Material

In this article, we assess the results of the proposed method across 6 different datasets drawn from various textual data extracted from information on social media. Each varies in dimension and characteristics because it contains important clues relating to depression, suicidal propensity, and other psychiatric attributes. This section provides substantial details about the structure and components of the datasets used during the classification process. The inclusion of six diverse datasets is a deliberate choice aimed at validating the robustness and generalizability of the proposed model. Depression and suicidal tendencies detection is highly context-dependent, and models trained on a single dataset may suffer from domain-specific biases. By incorporating datasets from different sources, platforms, and annotation schemes, we ensure that our model is capable of adapting to varied linguistic and psychological patterns. This comprehensive approach enhances the reliability of our findings, making the model more applicable in real-world mental health monitoring and intervention systems.

### 3.1. Dataset 1

The Suicidal Ideation Detection (SID) dataset (Ibtihaj, 2020) was designed to classify suicidal intent in tweet text based on the degree of seriousness. The dataset created contains tweets that mentioned the word suicide. They were labeled into three categories: Class 0—non-suicidal tweets (514), Class 1—tweets partially connected with suicidal intent (454), and Class 2—tweets indicating that a user may commit suicide (330).

**Figure 1:**
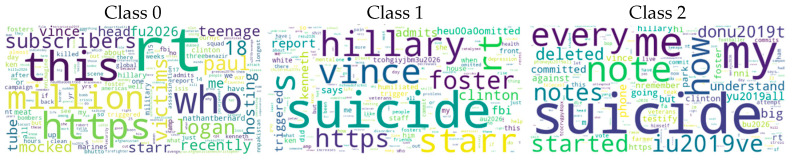
Word cloud representation for suicidal ideation detection categories.

Word maps were created to visualize important words belonging to each category in the dataset. The word maps in Figure 1 for Class 0, Class 1, and Class 2 show the most frequently used words in the tweet texts and their importance levels.

### 3.2. Dataset 2

**Figure 2:**
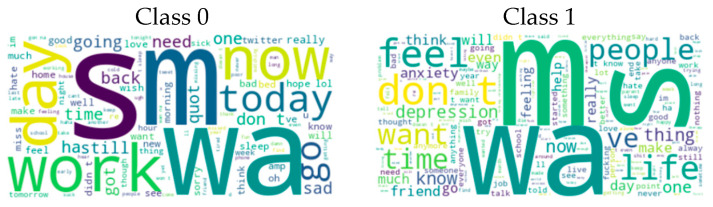
Word cloud representation for categories of Depression: Reddit Dataset.

Depression: The Reddit Dataset (Cleaned) (InFamousCoder, 2022) was collected from the Reddit platform using web scraping and cleaned using various natural language processing (NLP) techniques. The dataset, which consists of only English language texts, specifically targets mental health classification. The dataset is divided into two classes: Class 0 (content without depression symptoms, 3900 items) and Class 1 (content with depression symptoms, 3831 items). Figure 2 shows the word cloud generated for the classes in the Depression: Reddit Dataset.This dataset was obtained from the Kaggle platform (https://www.kaggle.com/datasets/infamouscoder/depression-reddit-cleaned, accessed on 10 November 2024) for use in studies on the detection and classification of texts with a depression content.

### 3.3. Dataset 3

**Figure 3:**
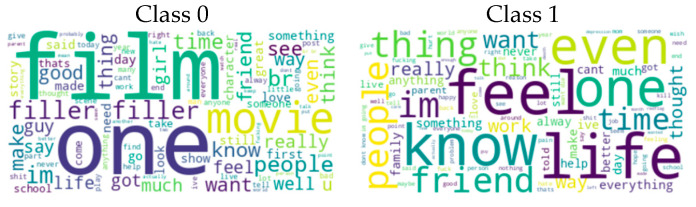
Word cloud representation for Mental Health Corpus Dataset categories.

The Mental Health Corpus dataset (Namdari, 2023) consists of texts related to anxiety, depression, and other mental health issues. It includes two columns: one containing comments and the other containing labels indicating whether the comments are toxic or not. This dataset serves multiple purposes, including sentiment analysis, toxic language detection, and the analysis of language related to mental health issues. It provides significant value to researchers, mental health practitioners, and others interested in studying language and emotions associated with mental health. Specifically, it plays an important role in identifying and analyzing toxic language linked to mental health. The dataset is divided into two categories: Class 0 (non-toxic comments, 14,139) and Class 1 (toxic comments, 13,838). Figure 3 shows the word cloud generated for the classes in the Mental Health Corpus dataset. The figure visualizes the most frequent words in Class 0 (non-toxic comments) and Class 1 (toxic comments). This visualization helps to understand language patterns and key terms used in discussions about mental health and also highlights salient terms that characterize toxic and non-toxic comments.

### 3.4. Dataset 4

**Figure 4:**
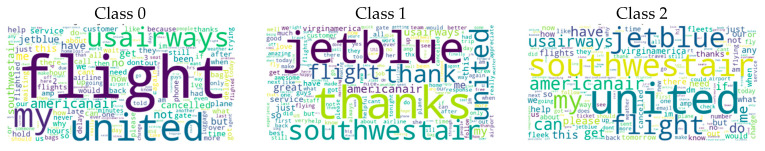
Word cloud representation for Twitter US Airline Sentiment dataset categories.

The Twitter US Airline Sentiment dataset (Kaggle, 2020) is taken from Crowdflower’s Data for Everyone library to perform sentiment analysis of tweets containing issues related to leading US airlines. This dataset consists of tweets collected during February 2015. Participants were asked to first classify the tweets as positive, negative, or neutral, and then to categorize the reasons for negative comments (e.g., “delayed flight” or “rude service”). The dataset is available on the Kaggle platform as a reorganized version of the original source. It is available both in a CSV format and as a SQLite database. The dataset presents the sentiment of tweets about the six major airlines in the US in the following classes: Class 0 (negative, 9178 tweets), Class 1 (positive, 3099 tweets), and Class 2 (neutral, 2363 tweets). Although Dataset 4 and Dataset 6 were originally annotated for sentiment analysis rather than the direct detection of depression and suicidal tendencies, prior studies have demonstrated a strong correlation between sentiment patterns and mental health conditions. Sentiment shifts, excessive negative expressions, and self-referential language are well-established indicators of psychological distress. Therefore, integrating these datasets enhances the robustness of our approach by capturing subtle linguistic cues associated with depression and suicidal tendencies. This broader dataset selection strengthens the model’s ability to generalize across diverse textual data, making it more applicable to real-world mental health assessments. Figure 4 shows the word cloud generated for the classes in the Twitter US Airline Sentiment dataset. The figure visualizes the most frequently used words in negative, positive, and neutral tweets. This visualization provides important clues to understand the language patterns used in tweets and the sentiment about airlines.

### 3.5. Dataset 5

**Figure 5:**
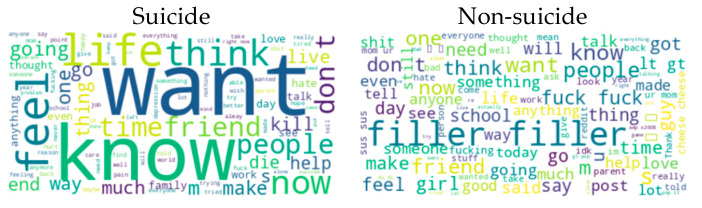
Word cloud representation for Suicide and Depression Detection dataset categories.

The Suicide and Depression Detection dataset (Komati, 2021) consists of texts collected from the “SuicideWatch” and “depression” subreddits on Reddit. The dataset was collected using the Pushshift API. From the “SuicideWatch” subreddit, all posts from 16 December 2008 (creation date) to 2 January 2021 were collected; from the “depression” subreddit, posts from 1 January 2009 to 2 January 2021 were included. Additionally, posts from the “r/teenagers” subreddit were labeled as normal (non-suicide) content. The dataset used in this study only consists of posts with suicide and non-suicide tags. For the analysis, 10,000 posts were selected from each tag. Figure 5 shows the word cloud generated for the classes in the Suicide and Depression Detection dataset. In the figure, the most frequent words in suicide and non-suicide posts are visualized. This visualization provides important insights into the language and expression patterns used in suicide ideation- and depression-themed posts.

### 3.6. Dataset 6

**Figure 6:**
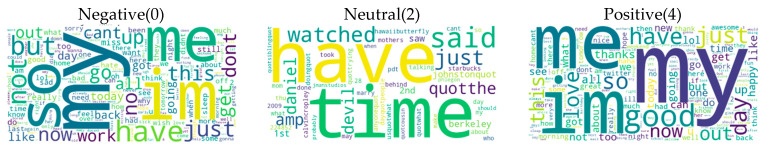
Word cloud representation for Sentiment140 dataset categories.

The Sentiment140 dataset (Go et al., 2009) consists of 1.6 million tweets obtained using Twitter API. The dataset is annotated to identify the sentiment of tweets and is widely used for sentiment classification. The labeling was carried out as follows: 0 (negative), 2 (neutral), and 4 (positive). In this study, 50,000 negative, 50,000 neutral, and 50,000 positive tweets were randomly selected for analysis using our proposed method. Figure 6 shows the word cloud generated for the classes in the Sentiment140 dataset. In the figure, the most frequent words in negative, neutral, and positive tweets are visualized. This visualization helps to understand the language and expression patterns used in tweets with different emotional states.

## 4. Method

**Figure 7:**
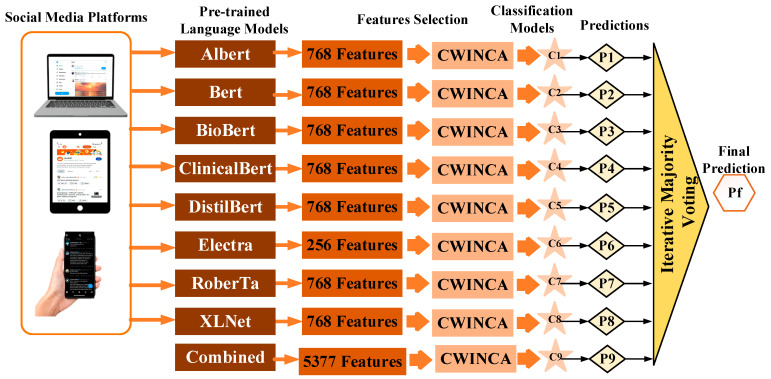
An overview of the proposed framework. The methodology involves (1) data preprocessing, (2) feature extraction using pre-trained language models, (3) feature selection via CWINCA, (4) classification using Support Vector Machines (SVMs), and (5) prediction aggregation using iterative majority voting (IMV). These steps collectively enhance the model’s accuracy and generalizability.

In this paper, we develop a comprehensive and robust framework for the classifi-cation and prediction of psychiatric conditions using textual data. The proposed methodology aims to achieve high accuracy and reliability by integrating pre-trained language models, feature selection techniques, and machine learning classifiers. The methodology is designed to address the challenges of text-based datasets such as high dimensionality, noise, and domain-specific word distributions. The overall architecture of the proposed model is depicted in Figure 7. It provides a schematic illustration of the methodology, outlining each step from raw text preprocessing to feature selection and final classification.

The methodology of the study consists of the following five steps:

Step 1: data preprocessing—cleaning and preparing raw datasets for analysis.

The raw text data were cleaned and normalized to improve model performance. In this process, stop words, punctuation marks, and special characters were removed from the texts; spelling errors were corrected and repetitive characters were normalized. In addition, the texts were broken down into smaller units (tokenization) and converted into a format that the models could process. Language processing techniques were applied according to the characteristics of each dataset and preparation for analysis was completed. These steps helped the model to learn meaningful patterns more efficiently by reducing the noise in the texts.

Step 2: feature extraction—extracting contextual features from texts using pre-trained language models.

Pre-trained language models were used to extract contextual features from the texts. These models include BERT, RoBERTa, ALBERT, DistilBERT, ClinicalBERT, etc. Each model generated contextual embeddings, representing the semantic content of the texts, and these attributes were combined to create a large set of attributes for analysis. The contextual embeddings generated by these pre-trained models were concatenated to form a comprehensive feature vector. This unified representation allowed the classifier to leverage the strengths of multiple language models simultaneously, providing a richer semantic understanding of the text data.

Step 3: feature selection—feature selection was performed separately for each model (ALBERT, BERT, BioBERT, ClinicalBERT, DistilBERT, ELECTRA, RoBERTa, XLNet) and for the combined feature vector derived from these models. This process aimed to eliminate redundant and irrelevant information from the high-dimensional feature space, thereby improving classification performance. To achieve this, Cumulative Weight-based Iterative Neighborhood Component Analysis (CWINCA) (Tuncer et al., 2024) was utilized to determine the importance of each feature. CWINCA iteratively assigns weights based on contributions to classification accuracy, ensuring that only the most relevant attributes are retained. The optimized feature sets generated for each model enhanced classification efficiency, accuracy, and interpretability by reducing noise and computational complexity. The detailed steps of the feature selection process are outlined in Algorithm 1.
**Algorithm 1.** Pseudocode of the Feature Selection Process.Input: Extracted features (X) with the size of R × C, Actual outputs (y) Output: Selected feature vector (SV)00: Load X 01: for j = 1 to C do 02:    //Normalize each column of X 03:     X(:, j) = (X(:, j) − min(X(:, j)))/(max(X(:, j)) − min(X(:, j)) + epsilon) 04: end for 05://Train feature selector model 06: mdl = fscnca(X, y, ‘Solver’, ‘sgd’) 07: xx = mdl.FeatureWeights 08://Rank features by importance 09: index = sort(xx, ‘descend’) 10: cumulativeWeights = cumsum(xx(index))/sum(xx(index)) 11://Determine starting and stopping points 12: startIndex = find(cumulativeWeights >= 0.5, 1) 13: if isempty(startIndex) then 14:     startIndex = 10 15: end if 16: stopIndex = find(cumulativeWeights >= 0.999, 1) 17: if isempty(stopIndex) then 18:     stopIndex = size(X, 2) 19: end if 20://Perform feature selection iteratively 21: bestLoss = inf 22: for ts = 0 to stopIndex − startIndex do 23:     for i = 1 to startIndex + ts do 24:         poz(:, i) = X(:, index(i)) 25:     end for 26:     27:    //Train k-NN model and calculate cross-validation loss 28:     mdl_knn = fitcknn(poz, y, ‘Distance’, ‘cityblock’, ‘NumNeighbors’, 1, ‘DistanceWeight’, ‘Equal’, ‘Standardize’, true, ‘ClassNames’, unique(y)’) 29:     kk = crossval(mdl_knn, ‘KFold’, 10) 30:     ll(ts + 1) = kfoldLoss(kk, ‘LossFun’, ‘ClassifError’) 31:     32:     clear poz 33: end for 34://Select best features 35: [bestLoss, inde] = min(ll) 36: for i = 1 to startIndex + inde − 1 do 37:     SV(:, i) = X(:, index(i)) 38: end for 39: Return SV

In addition to Support Vector Machines (SVMs), the study also evaluated the performance of k-Nearest Neighbors (k-NNs), Neural Networks, logistic regression, and Ensemble Learning methods. These baseline classifiers were included to provide comparative analysis and establish a performance benchmark for the proposed feature selection and extraction techniques.

Step 4: The selected features were then used to train and validate multiple classification models. To evaluate classification performance, SVM (Hearst et al., 1998), k-Nearest Neighbors (k-NNs), Neural Networks, logistic regression, and Ensemble Learning Methods were employed. SVM was trained with a polynomial kernel function to enhance accuracy. A 10-fold cross-validation strategy was implemented to assess the generalization capability of each classifier. Prediction vectors were recorded for each classification result to compare model performance, ensuring that the most effective approach was selected based on accuracy and robustness.

Step 5: Result integration—the predictions from the different models and feature sets were combined using an iterative majority voting method. We assigned each sample to the class most frequently predicted by the models, continuing this process until the final result was obtained. This increased overall performance by combining the strengths of different models. The CWINCA algorithm refined the feature space by iteratively updating feature weights based on their relevance to the target classes. This method helps to identify and retain the most meaningful attributes, improving classification accuracy while maintaining model interpretability. Additionally, iterative majority voting was used to aggregate predictions from different classifiers, enhancing the robustness of the final results by mitigating the effects of outlier predictions. This probably balanced the weaknesses of the models and resulted in more stable classification. Graphic analysis and visualizations complement the insights provided by majority voting to identify which model performs better. Furthermore, this approach significantly enhanced the reliability and validity of the proposed framework.

### Pre-Trained Language Models

The following pre-trained language models were used in this study to extract meaningful textual features from social media data. Each model was selected based on its strengths in handling natural language processing tasks:

**ALBERT (A Lite BERT):** ALBERT, introduced in 2019, is a lightweight language model designed to optimize the efficiency of BERT (Z. Lan, 2019). Through parameter factorization and cross-layer parameter sharing, ALBERT significantly reduces the number of parameters while maintaining a performance comparable to that of BERT, achieving this with 18 times fewer parameters (Choi et al., 2021; Z. Lan, 2019). The ALBERT model utilized in this study was trained on 40 GB of a 200 GB dataset, including free e-books, articles, newspapers, online blogs, Twitter, and Wikipedia. Its efficient architecture makes it a powerful choice for natural language processing tasks.

**BERT (Bidirectional Encoder Representations from Transformers):** BERT, introduced by Devlin et al. in 2018 (Devlin, 2018), is a bidirectional language representation model designed to pre-train deep representations on unlabeled text. Its architecture relies on a transformer encoder that utilizes the attention mechanism (Vaswani, 2017). By capturing both left and right contexts, BERT achieves high accuracy in various language modeling tasks.

**ClinicalBERT:** ClinicalBERT is a specialized adaptation of BERT optimized for clinical text analysis (Huang et al., 2019). Trained on extensive datasets of electronic health records (EHR) and clinical notes, it demonstrates superior performance in tasks such as medical text classification, sentiment analysis, and disease prediction.

**BioBERT:** BioBERT is tailored for biomedical text mining and pre-trained on large-scale biomedical corpora. It outperforms general-purpose models in tasks like named entity recognition, relation extraction, and question answering, making it particularly effective for biomedical applications (Lee et al., 2020).

**DistilBERT:** DistilBERT is a compact version of BERT created through model distillation techniques (Sanh, 2019). It retains most of BERT’s performance while being smaller and faster, offering an efficient alternative for tasks that require reduced computational resources (Adoma et al., 2020).

**RoBERTa:** Introduced in 2019, RoBERTa enhances BERT by eliminating the Next Sentence Prediction (NSP) task and implementing dynamic Masked Language Modeling (MLM) (Y. Liu, 2019). Trained on larger datasets with optimized configurations, RoBERTa delivers faster and more precise results compared to BERT.

**ELECTRA:** ELECTRA employs a substitution token detection approach instead of BERT’s Masked Language Modeling (MLM) technique (Clark, 2020). This innovation allows it to achieve higher accuracy while using fewer computational resources, making it an efficient solution for language understanding tasks.

**XLNet:** XLNet integrates autoregressive (AR) and autoencoder (AE) techniques, utilizing permutation-based training and a two-stream self-attention mechanism. These advancements enhance bidirectional context learning and enable XLNet to outperform BERT in language modeling tasks (Yang, 2019).

## 5. Experimental Results

This section presents the experimental results obtained from the proposed methodology. The evaluation includes a comparison of different classification models, an analysis of feature selection effectiveness, performance assessment across multiple datasets, and the impact of ensemble learning techniques.

### 5.1. Experimental Setup

The experiments were conducted using high-performance computing environments. Data preprocessing and feature extraction (Steps 1 and 2) were performed in Google Colab with high RAM capacity and an A100 GPU, enabling the efficient processing of large datasets. Feature selection, classification, and result integration (Steps 3, 4, and 5) were executed using MATLAB 2023 on a high-performance workstation (Windows 11 Pro, Intel i9 11th Gen processor, 128 GB RAM, 2 TB storage).

### 5.2. Classification Performance Across Models

To determine the most suitable classifier for the study, multiple machine learning algorithms were evaluated on the Suicidal Ideation Detection (SID) dataset. As presented in Table 1, Support Vector Machines (SVMs) consistently outperformed other classifiers, achieving an accuracy of 79.96% with BERT, while alternative models such as k-NNs (75.88%), Neural Networks (77.5%), and Ensemble Learning (77.42%) exhibited lower performance. Similarly, SVM achieved 79.27% accuracy with the combined feature set, further reinforcing its effectiveness in handling high-dimensional feature representations extracted from pre-trained language models. While logistic regression provided competitive results in some cases, its overall accuracy was lower, particularly when dealing with complex patterns in high-dimensional text data. This outcome suggests that logistic regression’s linear decision boundary may be insufficient to capture the nuanced relationships within the dataset. Similarly, decision trees showed lower performances, likely due to their tendency to overfit on certain data distributions, leading to reduced generalizability. These findings highlight the effectiveness of non-linear classifiers such as SVM in capturing intricate linguistic patterns associated with depression and suicidal tendencies.

### 5.3. Impact of Feature Selection (CWINCA)

**Figure 8:**
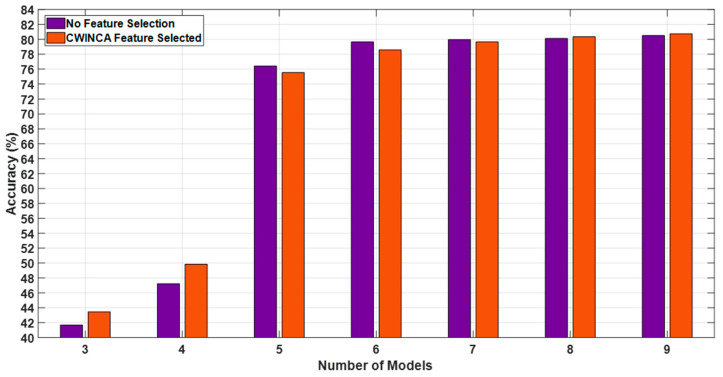
Accuracy comparison for Suicidal Ideation Detection with and without CWINCA feature selection.

To assess the effect of feature selection, we compared the classification accuracy with and without Cumulative Weight-based Iterative Neighborhood Component Analysis (CWINCA). Figure 8 demonstrates that CWINCA significantly improves classification performance, particularly when a limited number of models are employed. The results indicate that feature selection enhances generalization ability by eliminating redundant and irrelevant features, thereby reducing overfitting risks.

### 5.4. Performance Evaluation on Different Datasets

To validate the effectiveness of the proposed approach, experiments were conducted on six diverse datasets. The classification results were further analyzed using confusion matrices, which provide a detailed breakdown of model performance by illustrating the true positive (TP), false positive (FP), true negative (TN), and false negative (FN) rates for each dataset.

**Figure 9:**
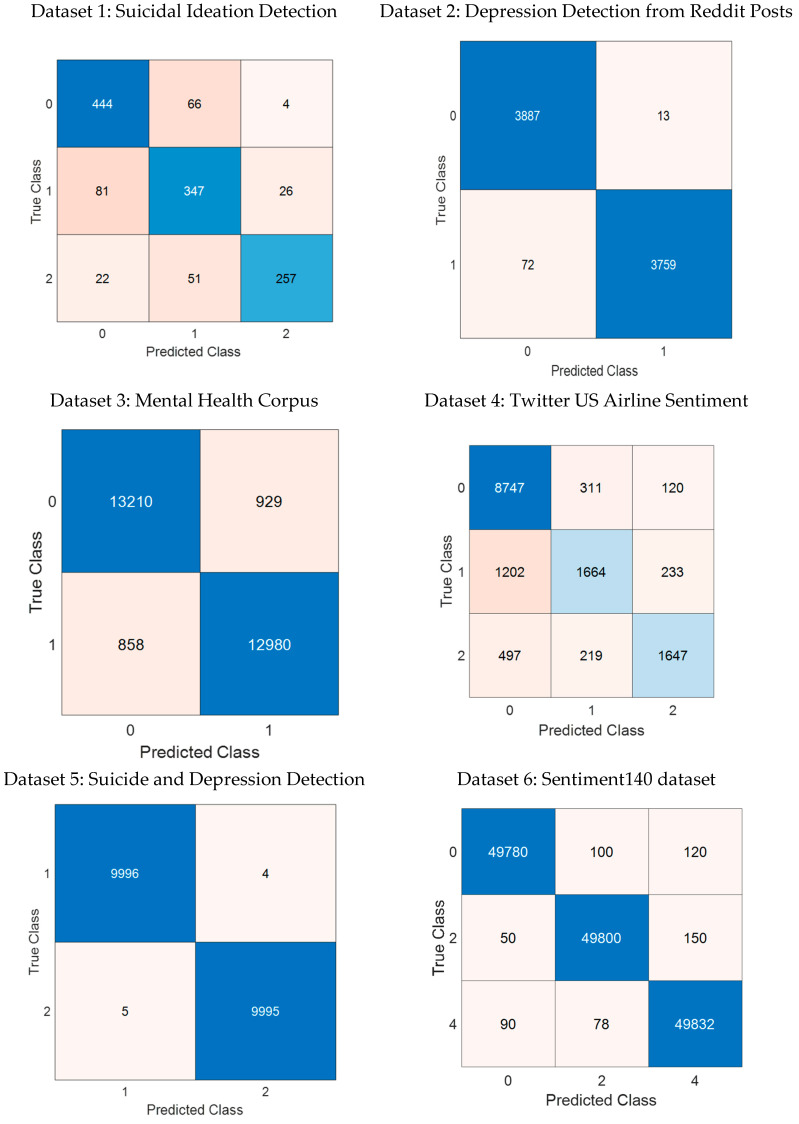
Confusion matrices for all datasets.

Figure 9 presents the confusion matrices for all datasets used in the study. These matrices visually depict how well the model distinguishes between different classes, offering insights into classification errors. The results indicate the following:

The model exhibits high precision and recall across datasets with balanced class distributions.

Misclassification rates are higher in datasets with class imbalance, such as Twitter US Airline Sentiment and SID datasets, where minority classes exhibit lower recall scores.

The Suicide and Depression Detection dataset achieved near-perfect classification accuracy, reinforcing the model’s robustness in well-structured datasets.

The confusion matrices further support the numerical results in Table 2, demonstrating the generalizability and effectiveness of the proposed framework. While certain datasets exhibit class imbalance challenges, the model’s feature selection and ensemble learning strategies help mitigate performance degradation, ensuring consistent classification accuracy.

### 5.5. Effect of Ensemble Learning (IMV Method)

**Figure 10:**
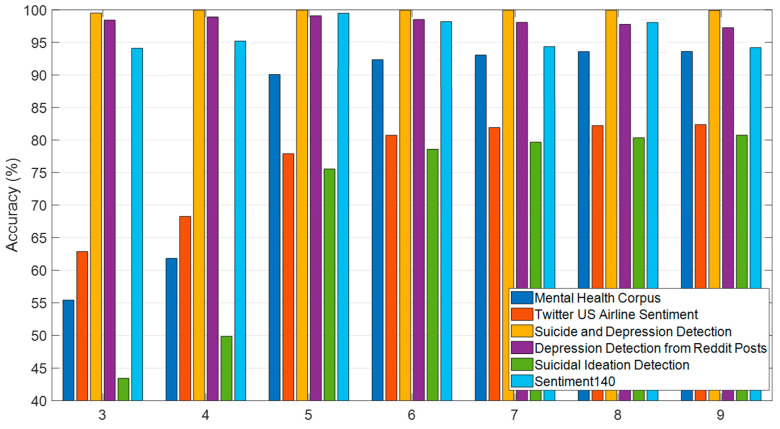
Accuracy comparison for IHMV results across datasets. (Dataset 1: SID dataset; Dataset 2: Depression: Reddit Dataset (Cleaned); Dataset 3: Mental Health Corpus dataset; Dataset 4: Twitter US Airline Sentiment dataset; Dataset 5: Suicide and Depression Detection dataset; Dataset 6: Sentiment140 dataset).

To further enhance classification accuracy, the iterative majority voting (IMV) method was applied, aggregating predictions from multiple models. As shown in Figure 10, IMV consistently improves accuracy across datasets. Notably, the Suicidal Ideation Detection dataset demonstrated a significant improvement, increasing from 43.45% accuracy with three models to 80.74% accuracy with nine models. These findings highlight the effectiveness of ensemble learning in improving classification robustness, particularly for complex and imbalanced datasets. The improvement in accuracy as the number of models increases from 3 to 9 in Figure 10 can be attributed to the Iterative Hybrid Majority Voting (IHMV) method, which enhances classification robustness by leveraging the diversity of multiple models. Unlike traditional majority voting, IHMV assigns different weights to models based on their individual performance, ensuring that more reliable models contribute more significantly to the final decision. As more models are incorporated, the ensemble benefits from a broader range of feature representations and decision boundaries, reducing the risk of individual model biases and improving generalization. Additionally, minority class recall tends to increase, as the ensemble effectively balances the strengths of different models, leading to a more comprehensive understanding of complex patterns in the data. However, beyond a certain number of models (typically after 9), performance gains plateau, indicating that additional models introduce redundancy rather than further improvements.

### 5.6. Discussion of Results and Key Findings

The experimental results demonstrate the effectiveness and generalizability of the proposed model. The key findings are summarized as follows:

- SVM consistently outperformed other classifiers, achieving the highest accuracy in almost all datasets.
- Feature selection using CWINCA significantly improved model performance by eliminating redundant attributes.
- The ensemble-based approach (IMV) further enhanced classification robustness, particularly in datasets with high class imbalance.
- The model demonstrated superior performance compared to existing methods, achieving the highest accuracy on the Suicide and Depression Detection dataset (99.96%).
- Despite minor performance fluctuations in certain datasets (e.g., lower recall for specific classes in the Twitter Sentiment dataset), the model maintained strong overall accuracy.

These findings confirm that the proposed approach effectively leverages pre-trained language models, feature selection techniques, and ensemble learning to enhance psychiatric disorder detection from social media data.

## 6. Discussion

A comprehensive discussion is crucial for understanding the strengths and limitations of the proposed method. This section evaluates the model’s performance in comparison to existing approaches, highlights its key contributions, and considers the clinical and computational implications of the findings.

### 6.1. Comparison with Existing Methods

This study compares the proposed method with various approaches in the existing literature on depression and suicidal ideation detection. The literature encompasses a wide range of machine learning and deep learning models, applied to diverse datasets, often demonstrating notable success. Table 3 summarizes the methods, data types, dataset sizes, results, and limitations of the models discussed in these studies. The proposed method addresses several challenges found in previous studies, particularly in terms of classification accuracy and robustness, through the integration of feature selection and model fusion techniques.

To further assess the effectiveness and generalizability of the proposed method, we examined its applicability to the datasets utilized in previous studies, as summarized in Table 3. While our approach is designed to be adaptable across various social media datasets, factors such as linguistic differences, annotation methodologies, and domain-specific characteristics may impact classification performance. Some of the datasets employed in prior research are publicly accessible, while others are subject to data-sharing restrictions, limiting direct re-evaluation. For the publicly available datasets, we intend to conduct additional experiments in future work to establish a direct performance comparison. This analysis will enable a more comprehensive assessment of our model’s robustness and its ability to generalize across diverse data sources. Furthermore, variations in data distributions and preprocessing strategies across different studies may influence model performance, necessitating the exploration of domain adaptation techniques to further enhance cross-dataset applicability.

The results presented in Table 3 demonstrate the superior performance and broader applicability of the proposed method compared to existing approaches for detecting depression and suicidal ideation in social media data. Notably, our framework achieved the highest accuracy rates on Dataset 5 (99.96%), Dataset 2 (98.9%), and Dataset 6 (99.61%), significantly outperforming previous methods such as BiLSTM-BiGRU (Faruq et al., 2024) and hybrid deep learning models (Tan et al., 2023; Tesfagergish et al., 2022). These findings highlight the effectiveness of integrating multiple pre-trained language models with advanced feature selection and ensemble learning techniques, which collectively enhance classification accuracy, robustness, and generalizability.

Many previous studies on depression and suicide risk detection from social media have been constrained by limited dataset diversity, reliance on specific platforms, and challenges in handling class imbalance. For example, approaches such as BiLSTM-BiGRU (Faruq et al., 2024) and BiLSTM, performed with Word2Vec/GloVe embeddings (Lestandy, 2023), rely exclusively on Reddit data, which, while informative, do not generalize well to other social media platforms such as Twitter, where language usage, user behavior, and content structure differ significantly. This platform dependency restricts the applicability of these models to broader, real-world mental health monitoring scenarios. Additionally, several previous studies use datasets that were originally designed for sentiment analysis rather than direct psychiatric disorder detection, which can lead to misclassification and reduced clinical validity. For instance, methods such as RoBERTa-GRU (Tan et al., 2023) and Tesfagergish et al.’s zero-shot ensemble model (Tesfagergish et al., 2022) leverage sentiment-labeled datasets like Sentiment140 and SemEval-2017. While there is a recognized correlation between sentiment patterns and mental health conditions, these datasets lack explicit annotations for psychiatric disorders, which may introduce biases and limit their effectiveness in detecting suicidal tendencies and clinical depression.

In contrast, our study mitigates these limitations by leveraging a diverse set of six datasets, including those explicitly focused on mental health (e.g., Suicide and Depression Detection, Suicidal Ideation Detection) as well as sentiment-based datasets, ensuring a more comprehensive evaluation of the model’s robustness across different data distributions. This multifaceted dataset approach enhances model generalizability, making it more suitable for real-world applications where input data characteristics vary significantly. Furthermore, the incorporation of Cumulative Weight-based Iterative Neighborhood Component Analysis (CWINCA) for feature selection significantly improves classification accuracy by eliminating redundant and less informative features, thereby enhancing the interpretability of predictions. Unlike prior methods that rely solely on complex deep learning architectures, our approach balances computational efficiency with predictive accuracy, making it more practical for real-time deployment.

The application of iterative majority voting (IMV) also plays a crucial role in enhancing classification robustness, particularly in handling class imbalance—a critical issue in psychiatric disorder datasets where the number of high-risk cases is often significantly lower than the number of non-risk cases. By integrating multiple model predictions, IMV reduces bias toward majority classes and improves the detection of less frequently occurring but clinically significant cases, ensuring a more equitable classification process. Given the promising results, the proposed framework presents strong potential for integration into real-world digital mental health monitoring systems. Unlike conventional sentiment analysis models, our approach is specifically tailored for psychiatric disorder detection, making it more aligned with clinical objectives. Future research should explore real-time applications of this method, extending its capabilities for continuous mental health monitoring and proactive intervention strategies. Additionally, incorporating multilingual datasets and cross-platform data sources could further enhance its global applicability, ensuring that AI-driven psychiatric disorder detection is inclusive and adaptable to diverse linguistic and cultural contexts.

In summary, our study demonstrates significant advancement over existing methods by addressing key limitations such as platform dependency, data diversity, and class imbalance. By integrating multiple transformer-based language models, robust feature selection mechanisms, and ensemble learning strategies, our method achieves a state-of-the-art performance while maintaining interpretability and computational efficiency, positioning it as a viable solution for large-scale, AI-driven mental health analysis.

### 6.2. Detailed Analysis of Performance Differences Across Models

While our study has demonstrated the superiority of the proposed approach over existing methods, it is essential to conduct a deeper analysis of the factors contributing to its enhanced performance. To achieve this, we conducted a detailed examination of feature representations at different network depths across various transformer-based models, including BERT, RoBERTa, ALBERT, and ClinicalBERT, using the SID dataset as the reference. This investigation aimed to uncover how different architectures encode suicidal ideation patterns and how feature extraction at different layers influences classification outcomes.

At the initial layers, ALBERT and DistilBERT, which utilize parameter-sharing and compression techniques to improve efficiency, generated compact feature representations. While this design reduces computational complexity, it may lead to information loss in the context of complex linguistic cues associated with suicidal ideation. In contrast, BERT and RoBERTa preserved richer word-level representations, effectively capturing subtle semantic and syntactic variations crucial for distinguishing high-risk instances from general discourse.

At the intermediate layers, RoBERTa exhibited enhanced contextual feature extraction compared to BERT, likely due to its dynamically masked language modeling strategy, which strengthens contextual embeddings. ClinicalBERT, pre-trained on clinical text, demonstrated superior alignment with psychiatric discourse, making it particularly effective for capturing mental health-related terminologies and expressions. However, its specialized nature resulted in slightly lower generalizability when applied to broader social media datasets.

In the final classification layers, the integration of Cumulative Weight-based Iterative Neighborhood Component Analysis (CWINCA) significantly refined feature selection by eliminating redundant or noisy attributes while preserving the most informative ones. This process contributed to a more robust classification pipeline, ensuring that the model prioritized relevant linguistic patterns indicative of suicidal ideation. Furthermore, the incorporation of iterative majority voting (IMV) improved classification stability by aggregating predictions from multiple transformer-based models, thereby mitigating potential biases and enhancing overall performance.

To provide further empirical validation, we quantitatively assessed the alignment of feature representations across different layers by measuring feature correlation across models. The results, presented in Table 4, illustrate how deep learning architectures encode information at various stages and how these representations contribute to the final classification performance. This comparative analysis underscores the advantages of leveraging a diverse ensemble of models with advanced feature selection and voting mechanisms, leading to improved accuracy, robustness, and generalizability in suicidal ideation detection.

### 6.3. Key Contributions of the Proposed Method

The proposed method integrates Cumulative Weight-based Iterative Neighborhood Component Analysis (CWINCA) for feature selection and Support Vector Machine (SVM) classifiers with pre-trained language models such as BERT, RoBERTa, and ClinicalBERT, offering significant advantages over existing approaches. The integration of CWINCA further optimized the computational process by eliminating redundant features, contributing to improved performance. Additionally, while methods like ABCDM (Basiri et al., 2021) and the MDI-Model (Wang et al., 2020) demonstrate limited performance on short texts, the proposed framework effectively handles both short and long texts, making it more adaptable to real-world scenarios.

### 6.4. Clinical and Psychological Implications

Beyond its computational advantages, our study has profound clinical implications for the early detection of depression and suicidal ideation. The ability to analyze social media data to identify individuals at risk of psychiatric disorders presents an opportunity for timely intervention and crisis management. However, it is essential to interpret AI-driven predictions within established psychiatric diagnostic frameworks, such as the DSM-5 criteria. To align our findings with established clinical standards, we conducted a linguistic pattern analysis, identifying critical indicators such as expressions of hopelessness, self-deprecating language, and reduced social engagement—hallmarks of depression and suicidality. These patterns align with the DSM-5 diagnostic criteria, reinforcing the clinical relevance of AI-based detection models. This suggests that automated mental health monitoring could serve as a valuable complementary tool for clinicians, aiding in early risk identification and intervention strategies. Despite its potential, the deployment of AI-driven mental health assessment systems must address key ethical challenges, including privacy concerns, data consent, and the risk of misclassification. False positives could lead to unnecessary distress or stigmatization, while false negatives could result in missed intervention opportunities. Therefore, future research should focus on integrating human-in-the-loop approaches, where AI models assist rather than replace clinical evaluations. Additionally, cross-disciplinary collaborations between AI researchers, mental health professionals, and policymakers will be essential to ensuring that these technologies are implemented ethically and responsibly. Such efforts could lead to the development of AI-assisted suicide prevention frameworks, incorporating digital interventions, real-time crisis alerts, and integration with professional mental health support services.

## 7. Conclusions

This study presents a novel approach for detecting depression and suicidal tendencies using social media data by integrating pre-trained language models and advanced feature selection techniques. The proposed methodology effectively extracts relevant features from high-dimensional, noisy social media text and optimizes classification performance through Cumulative Weight-based Iterative Neighborhood Component Analysis (CWINCA) and iterative majority voting. The experimental results demonstrate the robustness and generalizability of the model across six diverse datasets, emphasizing its potential for real-world mental health monitoring and intervention.

The findings highlight the efficacy of combining multiple pre-trained language models with optimized feature selection to enhance classification accuracy. Support Vector Machines (SVMs) consistently outperformed other classifiers, while CWINCA significantly improved interpretability by eliminating redundant features. Compared to existing approaches, our method achieves higher accuracy and better generalizability by leveraging a broader range of language representations. The ability to effectively handle imbalanced datasets further underscores the robustness of the proposed framework.

### 7.1. Limitations

Despite the promising results, the study has certain limitations that should be acknowledged. First, the model was trained primarily on English-language social media data, which may restrict its applicability to other linguistic and cultural contexts. Expanding the dataset to include multilingual sources will be necessary to improve global generalizability.

Second, class imbalance remains a challenge, as some datasets contain disproportionately fewer instances of high-risk cases. Although the iterative majority voting method helped mitigate this issue, additional balancing techniques, such as synthetic oversampling or adaptive weighting, could further enhance performance.

Third, the computational demands of the model present challenges for real-time applications. The reliance on multiple deep learning models and feature selection processes requires high-performance computing resources, limiting feasibility for large-scale, real-time deployment. Future work will focus on optimizing computational efficiency to make the model more practical for real-time depression and suicide risk detection.

Finally, while the model demonstrates high accuracy, further validation is required in clinical settings. Ensuring that the predictions align with psychiatric assessments and real-world mental health indicators is crucial for practical implementation. Future interdisciplinary collaborations with mental health professionals will be essential to refine and validate the model for real-world use.

### 7.2. Future Directions

To enhance model generalizability, future work will focus on expanding the dataset scope to include diverse linguistic and cultural representations. Advanced techniques for addressing class imbalance, such as synthetic data generation and adaptive re-weighting mechanisms, will be explored. Moreover, integrating next-generation pre-trained language models and refining the feature selection process will further improve classification accuracy and model interpretability.

An essential avenue for future research is the application of our proposed framework to additional benchmark datasets employed in previous studies. Conducting direct comparative analyses using these datasets would enable a more rigorous validation of our method’s performance and further substantiate its superiority over existing approaches. Given the availability constraints of certain datasets, our efforts will focus on those that are publicly accessible. Additionally, we will explore domain adaptation strategies to mitigate dataset-specific biases and improve the generalizability of the proposed model across varied linguistic and social media contexts.

Another key area for future development is the optimization of real-time applicability. Efforts will be directed toward reducing computational overhead to facilitate real-time depression and suicide risk detection in online environments. Additionally, the implementation of domain-specific language models tailored for mental health discourse may improve semantic understanding, leading to more precise and clinically relevant assessments.

Further validation through interdisciplinary collaboration with mental health professionals will also be prioritized. The integration of interpretable machine learning frameworks will enable a more transparent and clinically applicable approach, bridging the gap between computational psychiatry and practical mental health interventions.

This study contributes to the growing field of AI-driven mental health assessment by demonstrating the potential of social media data as a valuable resource for early risk detection. The proposed methodology advances the field by combining deep learning-based feature extraction with effective feature selection and classification techniques. Moving forward, improvements in model scalability, interpretability, and real-time application will further enhance the methodology’s utility for large-scale mental health monitoring and intervention systems.

## Figures and Tables

**Table 1:** Classification performance on the SID dataset.

	SVM	kNN	Tree	Neural Network	Efficient Logistic Regression	Ensemble
ALBERT	77.27	72.49	68.56	75.8	68.01	75.42
BERT	79.96	75.88	70.64	77.5	70.72	77.42
BioBERT	77.04	71.8	67.79	75.42	57.24	76.65
ClinicaBERT	76.57	71.41	68.95	74.88	57.01	75.8
DistilBERT	79.5	72.65	70.41	77.96	67.71	76.88
Electra	75.34	70.33	68.25	74.49	69.55	69.95
RoBERTa	79.5	72.72	70.33	77.73	58.92	78.35
XLNet	76.19	70.33	66.4	75.88	59.36	74.57
Combined	79.27	73.34	66.94	78.27	69.64	77.11

**Table 2:** Performance metrics of models across all datasets.

	Class	Accuracy (%)	Precision (%)	Recall (%)	F1Score (%)
Dataset 1	Class 1(0)	80.74	81.17	86.38	83.69
	Class 2(1)		74.78	76.43	75.60
	Class 3(2)		89.55	77.88	83.31
	Macro-Averaged		81.83	80.23	80.87
Dataset 2	Class 1(0)	98.90	98.18	99.67	98.92
	Class 2(1)		99.66	98.12	98.88
	Macro-Averaged		98.92	98.89	98.90
Dataset 3	Class 1(0)	93.61	93.90	93.43	93.66
	Class 2(1)		93.32	93.80	93.56
	Macro-Averaged		93.61	93.61	93.61
Dataset 4	Class 1(0)	82.36	83.74	95.30	89.15
	Class 2(1)		75.84	53.69	62.88
	Class 3(2)		82.35	69.70	75.50
	Macro-Averaged		80.64	72.90	75.84
Dataset 5	Class 1(1)	99.96	99.95	99.96	99.96
	Class 2(2)		99.96	99.95	99.95
	Macro-Averaged		99.96	99.96	99.95
Dataset 6	Class 1(0)	99.61	99.72	99.56	99.64
	Class 2(2)		99.64	99.6	99.62
	Class 3(4)		99.46	99.66	99.56
	Macro-Averaged		99.61	99.61	99.61

**Table 3:** Comparison of State-of-the-Art Methods.

Study	Method	Research Background and Objectives	Data Type Used	Number of Data	Limitations	Results
Faruq et al. (2024)	Combining BiLSTM and BiGRU with FastText embeddings for improved depression sentiment classification.	This study focuses on leveraging deep learning architectures to analyze depression-related Reddit posts and improve sentiment classification accuracy.	Reddit text data on depression and non-depression	7731 instances	The dataset is limited to Reddit, which may not generalize well to other social media platforms or clinical settings.	Accuracy = 97.03%, F1 score = 97.02%.
Lestandy (2023)	Utilizing BiLSTM with Word2Vec and GloVe embeddings to enhance feature representation in depression classification.	This research investigates the impact of different word embedding dimensions on the accuracy of depression classification models.	Depression: Reddit Dataset (labeled as depressed and non-depressed)	7731 instances; split as 75% training and 25% testing	The dataset lacks diversity and may be biased due to the informal language used on social media. Misclassification is possible due to contextual variations.	Accuracy = 96.22%, precision = 97.02%, recall = 95.30%, F1 score = 96.15%.
Cambria et al. (2024)	Implementing a neurosymbolic AI approach integrating hierarchical attention networks and commonsense knowledge representation.	The study introduces an interpretable AI model for sentiment analysis and suicidal ideation detection, emphasizing trustworthiness and explainability.	Sentiment140 (Twitter), First Impressions (personality), SuicideWatch (Reddit)	Polarity (1,440,144); personality (6000); suicide (138,479)	Interpretability of the models is limited. The use of SuicideWatch data raises ethical concerns regarding mental health predictions.	Accuracy = 99.34%.
Onan (2023)	Applying semantic role labeling (SRL) with ant colony optimization (ACO) for effective sentiment classification.	The research explores the effectiveness of semantic role labeling and ant colony optimization in improving sentiment classification models.	7 datasets: SST-2, Senti140, Yelp, US Airline, Toxic, SemEval, Sarcasm	Before: 1,795,666 (total); after: 3,613,637 (augmented)	Text augmentation may enhance model performance but can also introduce synthetic errors, reducing real-world applicability.	SST-2 accuracy = 89.17%, toxic = 99.45%.
Bendebane et al. (2023)	Developing a hybrid CNN-BiGRU deep learning model for multi-class classification of mental health conditions.	The study aims to classify normal, depression, and anxiety cases in Twitter data, particularly during the COVID-19 pandemic, using deep learning.	Twitter data during COVID-19	3,178,570 tweets	The dataset is specific to the COVID-19 period, limiting its generalization to post-pandemic social contexts.	Accuracy = 93.38%. F1 scores: normal = 96%, depression = 91%, anxiety = 93%.
Tan et al. (2023)	Combining RoBERTa transformer and GRU for enhanced sentiment analysis in various datasets.	This study evaluates the performance of a RoBERTa-GRU hybrid model in sentiment classification across diverse datasets.	IMDb, Sentiment140, Twitter US Airline datasets	IMDb: 50,000 reviews; Sentiment140: 1.6M tweets; Twitter: 14,160 tweets	The model is optimized for specific datasets, and its effectiveness in broader applications remains uncertain.	IMDb accuracy = 94.63%, Sentiment140 accuracy = 89.59%, Twitter US Airline accuracy = 91.52%.
Tesfagergish et al. (2022)	Using a two-stage model incorporating a zero-shot sentence transformer and an ensemble learning classifier for emotion detection.	The research focuses on enhancing emotion detection and sentiment classification through a zero-shot learning approach combined with ensemble learning.	IMDB, Sentiment140, SemEval-2017 datasets	IMDB: 50K reviews; Sentiment140: 1.6M tweets; SemEval-2017: 2.3K samples	The model struggles with multi-class classification, and dataset imbalances may impact performance.	Binary accuracy = 87.3%, F1 = 88.4%; Three-class accuracy = 62.7%, F1 = 55.4%.
Wang et al. (2020)	Proposing a sequential emotion analysis (SEA) and bidimensional hash search (BHS) model for mental disorder prediction.	The study aims to predict mental disorder severity using sequential emotion analysis and bidimensional hash search techniques.	Sentiment140, Twitter data (diagnosed-oriented and occupation-oriented), psychology blogs	Sentiment140: 1.04M tweets; blogs: 125K+, diagnosed data: 167K tweets	Diagnosis relies solely on textual data, which may lack clinical validation and lead to unreliable classifications.	Accuracy for diagnosed mental disorders: Anxiety (90.76%), Bipolar (90.70%), Depression (90.66%), OCD (90.44%).
Basiri et al. (2021)	Leveraging an attention-based bidirectional CNN-RNN deep learning framework for sentiment classification.	This research investigates how an attention-based CNN-RNN deep learning approach can improve sentiment classification on Twitter data.	Sentiment140 (Twitter dataset)	1.6M tweets (balanced positive/negative)	The model demonstrates relatively low accuracy, requiring further optimization for improved classification performance.	Accuracy: 81.82%, F1-Score (positive): 83.23%, F1-Score (negative): 80.76%.
Our Method	Integrating multiple pre-trained language models with Cumulative Weight-based Iterative Neighborhood Component Analysis (CWINCA) and iterative majority voting (IMV) for enhanced psychiatric disorder detection.	The study proposes a novel framework integrating deep learning and feature selection to enhance psychiatric disorder detection from social media data.	X (Twitter) and Reddit data (6 different datasets)	6 datasets, totaling millions of data points		Accuracy Dataset 1 = 80.74 Dataset 2 = 98.90 Dataset 3 = 93.61 Dataset 4 = 82.36 Dataset 5 = 99.96 Dataset 6 = 99.61

**Table 4:** Feature correlation of feature representations across different model layers for SID dataset.

Model	First Layer	Middle Layer	Last Layer	Final Output (SVM)
BERT	70.64	75.88	79.96	79.96
RoBERTa	70.33	72.72	79.50	79.50
ALBERT	68.56	72.49	77.27	77.27
ClinicalBERT	68.95	71.41	76.57	76.57
DistilBERT	70.50	76.50	79.50	79.50
Electra	68.25	70.33	75.34	75.34
XLNet	66.40	70.33	76.19	76.19
BioBERT	67.79	71.80	77.04	77.04
Combined	66.94	73.34	79.27	79.27

## Data Availability

In this paper, the dataset is publicly available.
